# Severe extra-glandular involvement and pleural effusions complicating primary Sjogren’s syndrome: a case report

**DOI:** 10.1186/s13256-022-03557-7

**Published:** 2022-10-18

**Authors:** Maen D. Abou Ziki, Roy Taoutel, Julian C. Hong, David N. Podell

**Affiliations:** 1 Department of Medicine, Yale University School of Medicine, 300 George St, New Haven, CT 06520 USA; 2 Department of Medicine, Lankenau Medical Center, Wynnewood, PA 19096 USA

**Keywords:** Primary Sjogren’s syndrome, Extra-glandular involvement, Biomarkers, Pleural effusion, Case report

## Abstract

**Background:**

Sjogren’s syndrome, an autoimmune disease of the exocrine glands, results in keratoconjunctivitis sicca, xerostomia, and dental caries. It is often overlooked, considered by clinicians to be a benign disease. However, it can cause life-threatening extra-glandular complications that affect multiple organ systems.

**Case presentation:**

Here we present a 78-year-old Caucasian woman with a history of primary Sjogren’s syndrome (pSS) whose symptoms of keratoconjunctivitis sicca were managed managed conservatively. She was evaluated for sub-acute shortness of breath. Imaging showed severe bronchiectasis with features of lymphocytic interstitial pneumonia. She also had exudative bilateral pleural effusions and skin ulcers, likely vasculitic in origin. The workup was significant for anti-Ro antibody, pancytopenia, hypocomplementia, cryoglobulinemia and monoclonal gammopathy, all of which reflect disease severity. Although there was no evidence of malignancy, she developed B-cell non-Hodgkin lymphoma during follow-up.

**Conclusions:**

Primary Sjogren’s syndrome can result in severe multi-organ disease. Pleural effusions are a rare complication of pSS, with only ten cases reported in the literature over the last 30 years, and tend to respond well to steroids. Prognostic biomarkers for disease severity include hypocomplementia, cryoglobulinemia, monoclonal gammopathy, and hypergammaglobulinemia. In this report we review the literature and the management of the disease.

## Background

Primary Sjogren’s syndrome (pSS) is estimated to affect 0.03–2.7% of the general population depending on the geographic location and the classification criteria used [1]. This autoimmune disease predominantly affects females with a 9:1 to 20:1 female:male ratio [1]. The most recent classification criteria for pSS, which were established by the American College of Rheumatology-European League Against Rheumatism (ACR-EULAR), include a list of five objective items as evidence of disease, with each item attributed a score. A total score of ≥ 4 out of 9 establishes the diagnosis of pSS. The criteria only apply to patients with at least one clinical manifestation of pSS, such as oral or ocular dryness, and include keratoconjunctivitis sicca with ocular staining, salivary gland biopsy with lymphocytic sialenditis (focus score of > 1 focus/mm^2^), antibodies positive for anti-SSA (Ro) and/or anti-SSB (La), positive test result for rheumatoid factor (RF) or anti-nuclear antibody (ANA), Schimer’s test indicative of decreased tear production, and decreased unstimulated salivary flow [2]. Of note, secondary Sjogren’s syndrome (sSS) refers to patients with an underlying autoimmune disease, such as systemic lupus erythematosis (SLE), rheumatoid arthritis (RA), mixed connective tissue disease, systemic sclerosis, or others and who concurrently develop Sjogren’s syndrome.

**Fig. 1:**
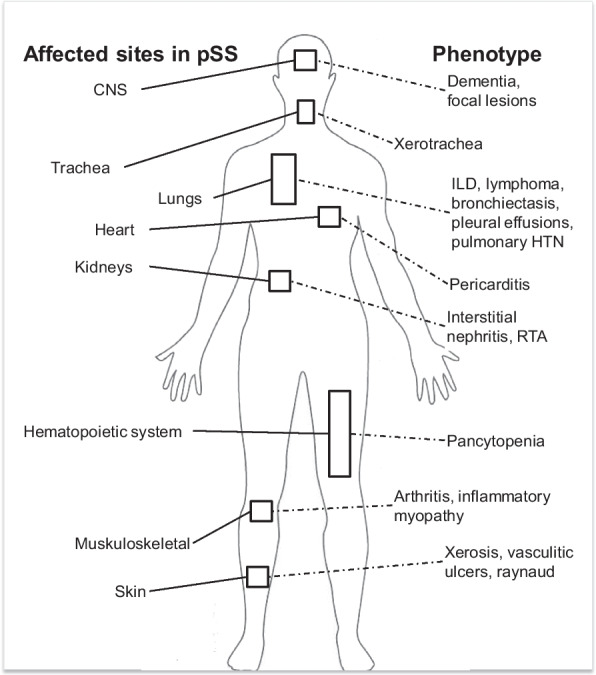
Summary of the disease manifestations in extra-glandular organs affected by primary Sjogren’s Syndrome (*pSS*).* CNS* Central nervous system,* HTN* hypertension,* ILD* Interstitial lung disease,* RTA* renal tubular acidosis

Sjogren’s syndrome is characterized by chronic inflammation and lymphocytic infiltration, but its pathogenesis is poorly understood. The concordance rate of pSS among monozygotic twins is only 20%, which signifies the role of epigenetics and environmental triggers [3]. In addition to the classical exocrine gland involvement, pSS can involve extra-glandular tissue, as highlighted in Fig. 1. Systemic involvement occurs in 10–20% of patients with pSS [4], and some of these complications can be fatal. Additionally, 4–9% of patients with pSS develop non-Hodgkins lymphoma [5, 6]. Several groups have attempted to identify serum markers that can predict disease severity as only a subset of patients develop severe extra-glandular complications.

Here we present a case of pSS with severe systemic disease that was complicated by pulmonary parenchymal involvement, bilateral pleural effusions, pancytopenia, cryoglobulinemia, hypocomplementia, and non-Hodgkin lymphoma.

## Case presentation

A 78-year-old Caucasian woman presented with sub-acute shortness of breath. She had a history of pSS diagnosed 10 years previously by lip biopsy and positive antibody markers. She had been managed conservatively for symptoms of eye and mouth dryness with artificial saliva, frequent sips of water, sugarless lemon drops, cyclosporin eye drops, and biodyne mouthwash and toothpaste. Her past medical history is significant for hypothyroidism (on levothyroxine 100 mcg), iron deficiency and anemia of chronic disease, bronchiectasis, complete heart block status post pacemaker, and remote history of breast cancer treated with tamoxifen and bilateral mastectomy over 20 years ago.

**Fig. 2:**
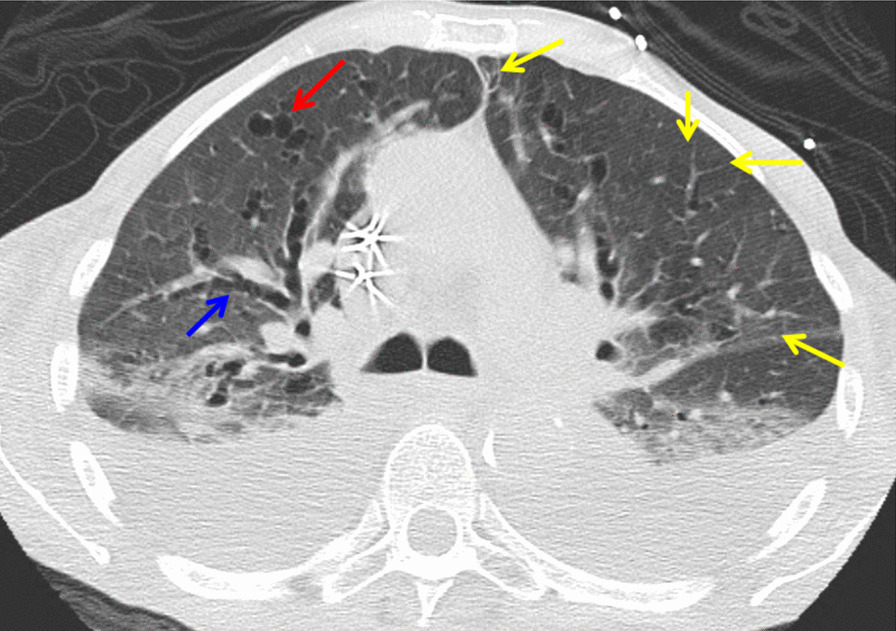
High-resolution computed tomography of the chest shows severe bronchiectasis (blue) bilaterally with septal thickening (yellow), and thin-walled cysts (red) consistent with radiographic features of lymphocytic interstitial pneumonia, a well-described interstitial lung disease in pSS. Bilateral pleural effusion was notable

She was noted to be tachypneic and hypoxemic, with 93% oxygen saturation on nasal cannula (5 L/minute) upon initial presentation. On examination she had dental caries and dry oral mucosal membranes. Moreover, there were diffuse ronchi and decreased breath sounds in her lower lobes bilaterally. Skin ulcers were noted on the shin of her lower extremity. High-resolution computed tomography of the chest showed large bilateral pleural effusions, severe bronchiectasis bilaterally with septal thickening, dilated medium-sized airways with eccentric pulmonary arteries, and thin walled cysts (Fig. 2).

The results of pleural fluid analysis were consistent with an exudate and were negative for signs of malignancy or infection. A pleural biopsy was also negative for malignant cells and the results were consistent with benign mesothelial cells and histiocytes. Echocardiography showed elevated right heart pressures (pulmonary arterial systolic pressure [PASP] of 39 mmHg), normal ejection fraction, mild left ventricular hypertrophy, and mild diastolic dysfunction. Additional laboratory test results showed pancytopenia: hemoglobin = 8.9 g/dL, white count = 3300/mm^3^, and platelets = 113,000/mm^3^. The red blood cell indices included an mean corpuscular volume (MCV) of 92.5 (reference range 78–100) fL and red cell distribution width (RDW) of 16% (reference range 11.5–14%). The results of the iron panel showed an iron level of 59 (reference range 60–170) mcg/dL, iron-binding capacity of 206 (reference range 240–450) mdg/dL, and ferritin of 29 (reference range 12–263) ng/mL. Vitamin B12 was 278 (reference range 160–950) pg/mL. The thyroid stimulating hormone (TSH) level was 4.770 (reference range 0.5–5.0) mIU/L, with normal free thyroxin (T4) at 1.66 (reference range 0.7–1.9) ng/dL. The vitamin D level was 45.1 (reference level 25–80) ng/mL. The rheumatology-targeted workup was significant for positive anti-Ro (SSA) antibody (> 8) (reference level: < 1), RF, elevated beta-2-microglobulin, cryoglobulinemia (level not quantitated), hypocomplementia (C3 < 83 [reference range 88–165] mg/dL; C4 < 8 [reference range 14–44] mg/dL), elevated inflammatory makers (erythrocyte sedimentation rate > 140 mm/hour; C-reactive protein = 44 mg/L), negative ANA, and monoclonal gammopathy of unknown significance (κ = 43.4 [reference range 0.33–1.94] mg/dL; λ = 31.1 [reference range 0.57–2.63] mg/dL; κ/λ =  1.4). Albumin level was normal.

The hypoxemia was likely multi-factorial, related to the large pleural effusions and parenchymal lung disease. Primary Sjogren’s syndrome explains her pleural effusions, bronchiectasis, and pulmonary hypertension as it causes inflammation and tissue injury in the pulmonary system. Additionally, the chest imaging results were consistent with radiographic features of lymphocytic interstitial pneumonia (LIP), which is a well-described interstitial lung disease (ILD) in Sjogren’s syndrome [7, 8].

**Fig. 3:**
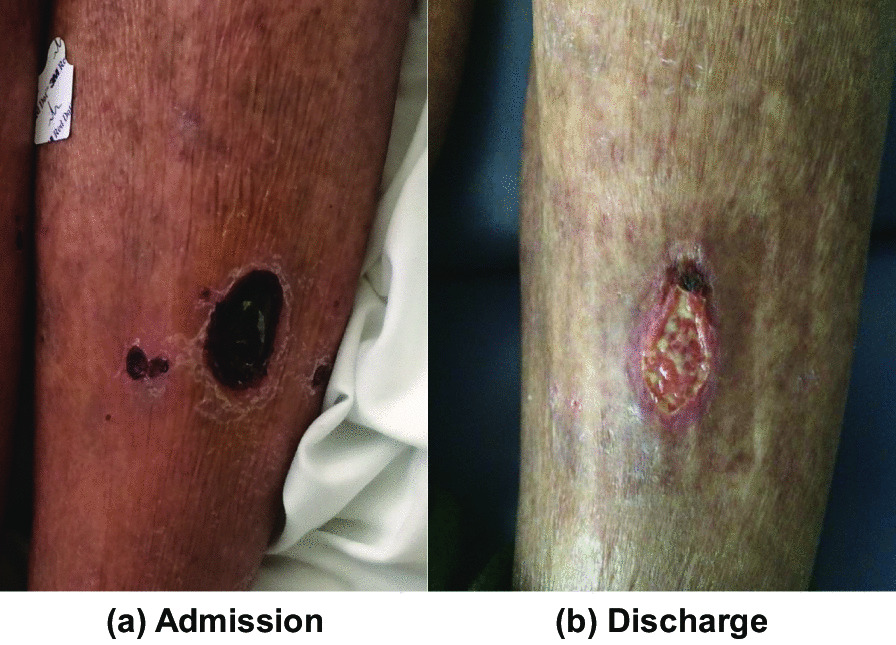
Skin ulcer on presentation (**a**) and well-healing ulcer after treatment (**b**)

A diagnosis of pSS with extra-glandular complications was made. She was started on prednisone 40 mg per day. Two chest tubes were placed to drain the effusion. At discharge the patient was feeling significantly better. Her shortness of breath had resolved. Moreover, her xerostomia, xerophthlamia, chronic fatigue, and appetite had improved. The skin ulcers had also started to heal. Although a punch biopsy was not performed, the skin ulcers are likely to be vasculitic as they were unprovoked and resolved after the initiation of prednisone (Fig. 3).

Prednisone was tapered after 6 weeks and the patient then switched to mycophenolate mofetil with continued improvement of her symptoms. Laboratory investigations demonstrated resolution of the hypocomplementia, cryoglobulins, and improvement in all cell lines. Inflammatory markers and beta-2-microglobulin trended downward. However, the anemia was persistent and she was started on darbepoetin alfa.

At 5 months of follow-up, the patient was noted to have a swollen submandibular lymph node, which was surgically excised. Pathology revealed a B-cell marginal zone lymphoma (CD19, CD45, and IgM-positive immunophenotype).

## Discussion

Sjogren’s syndrome is very often overlooked as a benign disorder. However, it can cause multi-organ involvement and life-threatening pulmonary disease. While pulmonary involvement is a commonly seen extra-glandular manifestation in 5–12% of patients with pSS [4], there have only been ten cases of pSS with pleural effusion reported in the literature. More commonly, pulmonary involvement manifests as bronchiectasis, xerotrachea, ILD, lymphomas, and pulmonary hypertension.

The first case of pSS with pleural effusion was reported in 1989, and over the last 26 years there has been only ten published case reports (Table 1). Consistent with the literature, the pleural effusions in our patient were exudative and responded to immunosuppression. Although no longitudinal follow-up was found on the few reported cases, it is likely that pleural effusions are an indicator of aggressive disease. Prognostic markers that are associated with severe extra-glandular disease include hypocomplementia (C3 and C4), cryoglobulinemia, monoclonal gammopathy, anti-Ro, anti-La, RF, and hypergammaglobulinemia, most of which were also present in our patient [5, 6, 9, 10].

**Table 1:** Characteristics of patients with primary Sjogren’s syndrome with pleural effusions in the literature.

Phenotype	*N*	Gender	Age (years)	Skin involvement	Pleural fluid analysis	CT imaging	Cell line defect	Prednisolone dose (mg/day)	Response to steroids	Reference
Pleural effusion	1	Female	64	Petechia	Exudate	Effusion only	No	None	n/a	[11]
	1	Male	62	None	Exudate	Effusion only	Anemia	40	Resolved	[12]
	1	Female	40	Erythema	Exudate	No CT	Anemia	60	Resolved	[13]
	1	Male	70	None	Exudate	No CT	Anemia, and thrombocytopenia	30	Recurred	[14]
	1	Female	45	Petechia	Exudate	No CT	Anemia	Unknown	Resolved	[15]
	1	Male	65	None	Exudate	Effusion only	No	60	Resolved	[16]
Pleural and pericardial effusion in pregnancy	1	Female	35	None	No tap	No CT	Anemia	30	Resolved	[17]
Pleural effusion with diabetic nephropathy	1	Male	73	None	Exudate	Effusion only	Anemia	30	Failed	[18]
Pleural effusion and type II mixed cryoglobulinemia	1	Female	53	Petechia	Exudate	Effusion only	Pancytopenia	Prednisone, AZA and cytoxan	Resolved	[19]
Pleural effusion with sarcoidosis	1	Female	51	None	No tap	Effusion and hilar lymphadenopathy	No	None	n/a	[20]
Total	10									

*N* Number of patients, *CT* computed tomography of the chest, *AZA* azathioprine,* n/a* not applicable

Interestingly, although pSS is more common in women, available reports do not suggest any gender predilection in those cases complicated by pleural effusions (Table 1).

Various cutaneous manifestations have been reported in pSS, including skin dryness, maculopapular rash, annular erythema, and vasculitis [21, 22]. The incidence of vasculitis in patients with pSS is thought to be secondary to complement activation by the anti-SSA/SSB antibodies and occurs in around 9% of cases [21, 22]. It usually presents as palpable or non-palpable purpura that mainly affects the lower extremities [21, 22]. In addition, cutaneous ulcers have been described in up to 8% of patients [22]. The incidence of pSS-induced vasculitis is higher in patients who are positive for RF factor, and up to one third of patients are found to have cryoglobulinemic vasculitis. It is believed that lack of cryoglobulin detection accounts for the remaining cases [22]. In fact, the association between pSS and cryoglobulinemic vasculitis is well-established in the literature and is associated with a worse outcome [21]. Additionally, leukocytoclastic vasculitis, described in type II and III cryoglobulinemia, has been reported in 90% of the histopathological examinations of cutaneous Sjogren’s lesions [22].

Finally, patients with pSS have up to a 40-fold increased risk for lymphoma, with a lifetime risk of 5%. Cutaneous vasculitis occurs in about 10% of patients with pSS [6]. It was observed that cutaneous vasculitis, low C3 and C4 levels, cryoglobulinemia, low CD4 to CD8 ratio, persistent parotid gland enlargement, splenomegaly, and lymphadenopathy are significant risk factors for the development of non-Hodgkin lymphoma [10]. Therefore, clinicians should to be vigilant in their surveillance for lymphomas and pseudo-lymphomas in patients with pSS with extra-glandular manifestations and the aforementioned risk factors.

## Conclusions

Primary Sjogren’s syndrome presents with benign symptoms in the majority of cases; however, it can cause severe systematic disease that can be fatal. Research efforts to risk stratify patients have focused on biomarkers, with promising results. Pleural effusions are rare complications in patients with pSS and could signify severe extra-glandular disease. The effusions seem to respond well to steroid pharmacotherapy. Finally, patients with pSS have a very high risk of lymphoma; therefore, clinicians need to conduct vigilant screening, especially in patients with cutaneous vasculitis, low complement levels, crygloobulinemia or signs of severe disease.

## Data Availability

Data sharing is not applicable to this article as no datasets were generated or analysed during the current study.

## Abbreviations

ANA: Anti-nuclear antibody
ILD: Interstitial lung disease
MCV: Mean corpuscular volume
PASP: Pulmonary artery systolic pressure
pSS: Primary Sjogren’s syndrome
RA: Rheumatoid arthritis
RF: Rheumatoid factor
RDW: Red cell distribution width
SLE: Systemic lupus erythematosus
sSS: Secondary Sjogren’s syndrome

## References

[1] Patel R, Shahane A. The epidemiology of Sjogren’s syndrome. Clin Epidemiol. 2014;30(6):247–55. 10.2147/CLEP.S47399 PMC4122257 25114590

[2] Shiboski CH, Shiboski SC, Seror R, *et al*. 2016 American College of Rheumatology/European League Against Rheumatism Classification Criteria for Primary Sjögren’s Syndrome: a consensus and data-driven methodology involving three international patient cohorts. Arthritis Rheumatol. 2017;69(1):35–45. 10.1002/art.39859. PMC5650478 27785888

[3] Thabet Y, Le Dantec C, Ghedira I, Devauchelle V, Cornec D, Pers JO, *et al*. Epigenetic dysregulation in salivary glands from patients with primary Sjogren’s syndrome may be ascribed to infiltrating B cells. J Autoimmun. 2013;41:175–81. 10.1016/j.jaut.2013.02.002 23478041

[4] Baldini C, Pepe P, Quartuccio L, Priori R, Bartoloni E, Alunno A, *et al*. Primary Sjogren’s syndrome as a multi-organ disease: impact of the serological profile on the clinical presentation of the disease in a large cohort of Italian patients. Rheumatology (Oxford). 2014;53(5):839–44. 10.1093/rheumatology/ket427 24369420

[5] Solans-Laque R, Lopez-Hernandez A, Bosch-Gil JA, Palacios A, Campillo M, Vilardell-Tarres M. Risk, predictors, and clinical characteristics of lymphoma development in primary Sjogren’s syndrome. Semin Arthritis Rheum. 2011;41(3):415–23. 10.1016/j.semarthrit.2011.04.006 21665245

[6] Voulgarelis M, Ziakas PD, Papageorgiou A, Baimpa E, Tzioufas AG, Moutsopoulos HM. Prognosis and outcome of non-Hodgkin lymphoma in primary Sjogren syndrome. Medicine (Baltimore). 2012;91(1):1–9. 10.1097/MD.0b013e31824125e4 22198497

[7] Taouli B, Brauner MW, Mourey I, Lemouchi D, Grenier PA. Thin-section chest CT findings of primary Sjogren’s syndrome: correlation with pulmonary function. Eur Radiol. 2002;12(6):1504–11. 10.1007/s00330-001-1236-7 12042961

[8] Uffmann M, Kiener HP, Bankier AA, Baldt MM, Zontsich T, Herold CJ. Lung manifestation in asymptomatic patients with primary Sjogren syndrome: assessment with high resolution CT and pulmonary function tests. J Thorac Imaging. 2001;16(4):282–9. 10.1097/00005382-200110000-00009 11685093

[9] Skopouli FN, Dafni U, Ioannidis JP, Moutsopoulos HM. Clinical evolution, and morbidity and mortality of primary Sjogren’s syndrome. Semin Arthritis Rheum. 2000;29(5):296–304. 10.1016/s0049-0172(00)80016-5 10805354

[10] Theander E, Henriksson G, Ljungberg O, Mandl T, Manthorpe R, Jacobsson LT. Lymphoma and other malignancies in primary Sjogren’s syndrome: a cohort study on cancer incidence and lymphoma predictors. Ann Rheum Dis. 2006;65(6):796–803. 10.1136/ard.2005.041186 PMC1798187 16284097

[11] Alvarez-Sala R, Sanchez-Toril F, Garcia-Martinez J, Zaera A, Masa JF. Primary Sjogren syndrome and pleural effusion. Chest. 1989;96(6):1440–1. 10.1378/chest.96.6.1440b 2582863

[12] Ogihara T, Nakatani A, Ito H, Irokawa M, Ban S, Takahashi A, *et al*. Sjogren’s syndrome with pleural effusion. Intern Med. 1995;34(8):811–4. 10.2169/internalmedicine.34.811 8563128

[13] Kashiwabara K, Kishi K, Narushima K, Nakamura H, Yagyu H, Kiguchi T, *et al*. Primary Sjogren’s syndrome accompanied by pleural effusion. Nihon Kyobu Shikkan Gakkai Zasshi. 1995;33(11):1325–9. 8583730

[14] Kawamata K, Haraoka H, Hirohata S, Hashimoto T, Jenkins RN, Lipsky PE. Pleurisy in primary Sjogren’s syndrome: T cell receptor beta-chain variable region gene bias and local autoantibody production in the pleural effusion. Clin Exp Rheumatol. 1997;15(2):193–6. 9196874

[15] Tanaka A, Tohda Y, Fukuoka M, Nakajima S. A case of Sjogren’s syndrome with pleural effusion. Nihon Kokyuki Gakkai Zasshi. 2000;38(8):628–31. 11061090

[16] Teshigawara K, Kakizaki S, Horiya M, Kikuchi Y, Hashida T, Tomizawa Y, *et al*. Primary Sjogren’s syndrome complicated by bilateral pleural effusion. Respirology. 2008;13(1):155–8. 10.1111/j.1440-1843.2007.01149.x 18197929

[17] Mutsukura K, Nakamura H, Iwanaga N, Ida H, Kawakami A, Origuchi T, *et al*. Successful treatment of a patient with primary Sjogren’s syndrome complicated with pericarditis during pregnancy. Intern Med. 2007;46(14):1143–7. 10.2169/internalmedicine.46.0062 17634717

[18] Horita Y, Miyazaki M, Kadota J, Watanabe T, Yamashita M, Nishiura K, *et al*. Type II diabetes mellitus and primary Sjogren’s syndrome complicated by pleural effusion. Intern Med. 2000;39(11):979–84. 10.2169/internalmedicine.39.979 11065256

[19] Suzuki H, Hickling P, Lyons CB. A case of primary Sjogren’s syndrome, complicated by cryoglobulinaemic glomerulonephritis, pericardial and pleural effusions. Br J Rheumatol. 1996;35(1):72–5. 10.1093/rheumatology/35.1.72 8624627

[20] Tokuyasu H, Harada T, Touge H, Kawasaki Y, Maeda R, Isowa N, *et al*. Primary Sjogren’s syndrome complicated by sarcoidosis. Intern Med. 2008;47(23):2049–52. 10.2169/internalmedicine.47.1526 19043259

[21] Fox RI. Extraglandular manifestations of Sjögren’s syndrome (SS): dermatologic, arthritic, endocrine, pulmonary, cardiovascular, gastroenterology, renal, urology, and gynecologic manifestations. In: Fox R, Fox C, editors. Sjögren’s syndrome. New York: Springer; 2011. p. 285–316. 10.1007/978-1-60327-957-4_17.

[22] Generali E, Costanzo A, Mainetti C, Selmi C. Cutaneous and mucosal manifestations of Sjögren’s syndrome. Clin Rev Allergy Immunol. 2017;53(3):357–70. 10.1007/s12016-017-8639-y. 28871434

